# Minor Allele Frequency Filtering Can Strongly Bias Uniqueness Estimates Within and Across Species

**DOI:** 10.1002/ece3.73314

**Published:** 2026-03-24

**Authors:** Yuchi Zheng

**Affiliations:** ^1^ Chengdu Institute of Biology, Chinese Academy of Sciences Chengdu China

**Keywords:** across‐species estimate, genetic distance, MAF filter, RAD‐seq SNP, rare allele, uniqueness

## Abstract

Minor allele frequency filtering is a common practice in single‐nucleotide polymorphism (SNP) dataset construction. It can remove not only potential genotyping errors but also young, local, introgressed, and rare alleles critical to inferring some particular processes or variables, but the effects remain unknown for many downstream analyses. Among them, the evaluation of genetic uniqueness, a fundamental genetic parameter frequently considered in conservation planning and in breeding, may be susceptible because infrequent alleles are relatively unique. Using genome‐wide SNPs of two anurans and based on between‐individual genetic distances calculated by different methods, with a 0.5‐degree grid, I explored the effect of global minor allele frequency filtering (1%, 2%, 5%, and 10%) on within‐ and across‐species uniqueness estimates. At both levels, the deviation from estimates based on unfiltered data increased with the filtering strength, being notable mostly at thresholds of 5% and 10%, for example, a ~750 km shift of the grid cell with the most unique local population. Such impacts were not consistent across species. The effect may depend partly on the percentage of samples that are most genetically unique, so a low threshold does not necessarily guarantee high reliability. In addition, the comparison implies that a relatively unique population can appear to be genetically highly typical (i.e., representative) at higher thresholds. This study provides early evidence for considering a potential adverse effect of minor allele frequency filtering when obtaining and interpreting uniqueness estimates. It is among those calling for further investigation on the effects of parameters and filters involved in the creation of high‐throughput sequencing datasets.

## Graphical abstract

This study provides early evidence of minor allele frequency filtering biasing uniqueness estimates. Low filtering thresholds (e.g., 1%) do not necessarily guarantee high reliability. Relatively unique populations can appear to be typical at thresholds of 5% and 10%.

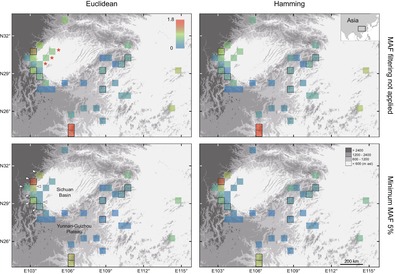

## 1 Introduction

Rare alleles in a SNP dataset can be caused by sequencing errors and can cause confounded inferences (Johnson and Slatkin 2008; Linck and Battey 2019). A minimum minor allele frequency (MAF) filter of up to 10% has often been suggested and commonly applied before analysis (e.g., Lou et al. 2021; Maffey et al. 2022; Bazakos et al. 2023; Schmidt et al. 2024; Vega‐Sánchez et al. 2024). This usually excludes a significant or even major portion of loci, given the prevalence of low‐frequency variants (e.g., van den Berg et al. 2020; Hall et al. 2020; Fu et al. 2021; Clark et al. 2022), despite the fact that a reduced error rate of next‐generation sequencing (Stoler and Nekrutenko 2021) and genotyping quality control can also improve the signal‐to‐noise ratio of the data. Frequently discussed together with SNP ascertainment bias, the effect of minor allele filtering has only been explored for some types of analysis, and it is not necessarily positive (e.g., Tabangin et al. 2009; la Cruz and Raska 2014; Malomane et al. 2018; O'Leary et al. 2018; Dokan et al. 2021; Lou et al. 2021; Pearman et al. 2022; Arantes et al. 2023; Hemstrom et al. 2024; Rick et al. 2024). Among the other analyses, the evaluation of genetic uniqueness may be particularly sensitive to this frequently used filtering (e.g., Zhao et al. 2020; Machová et al. 2023) because infrequent alleles are relatively unique.

Genetic uniqueness, though sometimes considered overemphasized, is a fundamental genetic parameter and one of the important criteria for conservation and breeding (Coleman et al. 2013; Weeks et al. 2016; Ralls et al. 2018; Nielsen et al. 2020; Fernandez‐Fournier et al. 2021; Willi et al. 2022; Schmidt et al. 2024). Like other aspects of intraspecific diversity, when inferred from a large number of genome‐wide SNPs, it is typically based on sampling across many localities, each usually with a small number of individuals, and on an overall minor allele filter if MAF filtering is applied (e.g., Hendricks et al. 2017; Linck and Battey 2019; DeWoody et al. 2021; Portanier et al. 2023; Ada et al. 2024; Hemstrom et al. 2024). Using pairwise genetic distances among individuals, clustering methods such as neighbor‐joining tree construction and principal coordinates analysis provide a qualitative evaluation of uniqueness. As a simple and straightforward quantitative evaluation, one can use corresponding averages calculated from these distances (Eding and Laval 1999). For instance, for each locality within a given grid cell, an average genetic distance to samples of other grid cells can be obtained, with the highest one assigned as an estimate of uniqueness for the grid cell. Such regional estimates for different species may further be normalized and combined (e.g., by adding) in detecting spatial patterns of diversity.

This raises the possibility of minor allele filtering affecting uniqueness estimates above the species level. It is not uncommon for different species to have endemic lineages associated with the same geographical area (Rincon‐Sandoval et al. 2019; Marske et al. 2020; Thomaz and Knowles 2020). For each of these species, the relative uniqueness estimates for populations of the endemic lineage might increase to some extent after excluding loci with the rarest alleles found elsewhere. In such a case, there may even be a considerable increase in the combined uniqueness estimate for the area or an overlapping grid cell.

The Emei mustache toad *Leptobrachium boringii* (Megophryidae) and spiny‐bellied frog *Quasipaa boulengeri* (Dicroglossidae) constitute a suitable system for exploring the effect of minor allele filtering on uniqueness estimation. These montane frogs occur in the subtropical East Asian mainland with overlapping distributions largely around the Yunnan‐Guizhou Plateau, including part of the low‐elevation eastern edge of the Mountains of Southwest China biodiversity hotspot (Fei et al. 2012; Hoffman et al. 2016; Fei 2020; Yan et al. 2021). The edge has been proposed to provide refugia or areas of long‐term persistence for various plant and animal species (e.g., Qi et al. 2012; Ye et al. 2014; Zheng et al. 2020). A previous analysis of data from restriction site‐associated DNA sequencing (RAD‐seq) has detected a *L. boringii* lineage corresponding to it, and this is also the case for *Q. boulengeri* (Zheng et al. 2020, 2025).

In the present study, using RAD‐seq SNPs of these frogs and based on between‐individual genetic distances calculated by different methods, with a 0.5‐degree grid, I explore the effect of MAF filtering on the estimation of uniqueness within and across species. The results at both levels show significant changes for some grid cells after applying an overall MAF filter of, for example, 5%, making the case that a potential effect of such filters should be considered when generating and interpreting genetic uniqueness estimates.

## 2 Materials and Methods

### 2.1 Genome‐Wide SNP Data

The published RAD‐seq data of *Leptobrachium boringii* (BioProject PRJNA551927; Zheng et al. 2020) and *Quasipaa boulengeri* (doi.org/10.57760/sciencedb.14822; Zheng et al. 2025) were used to identify SNPs. They were generated in Novogene (Beijing, China) using SLAF library construction (Sun et al. 2013) and Illumina platforms with relatively low sequencing error rates in PE150 mode, that is, Hiseq 2000 for *L. boringii* and Novaseq 6000 for *Q. boulengeri* (Stoler and Nekrutenko 2021; Euformatics comparer of sequencing platforms at https://q.omnomics.com/ords/f?p=118:34). Their sampling followed a common strategy in the era of high‐throughput sequencing: many sampling sites with a small number of individuals per site (e.g., Linck and Battey 2019; Portanier et al. 2023). The former contained 75 individuals from 25 localities across 18 half‐degree grid cells, mostly 3 individuals per locality. The latter comprised 55 pure *Q. boulengeri* individuals of an inferred hybridogenetic complex (Zheng et al. 2025), from 36 localities across 28 half‐degree grid cells with typically 1–2 individuals per locality. These localities were defined following the original publications (Table S1). They were distributed throughout the ranges of the two species, between which nine grid cells were shared. The 25 *L. boringii* localities represented all the known disjunct distributions of the species (Zheng et al. 2020). *Q. boulengeri* has a more continuous distribution range, with most of it represented by the 36 sampling localities (Yan et al. 2021; Zheng et al. 2025).

FastQC version 0.11.9 (Andrews 2010) was used to check the quality of reads. The removal of reads with adapter sequences was conducted by Novogene. Further cleaning was performed using the process_radtags module of Stacks version 2.66 (Catchen et al. 2013), excluding reads with any uncalled bases or 22‐bp sliding windows showing an average Phred33 score below 10.

In the absence of conspecific genome assemblies, *de novo* assembly of loci was conducted with the Stacks denovo_map.pl pipeline (Paris et al. 2017). R2 reads were treated as R1 reads. For SLAF RAD‐seq, the forward (or reverse) reads of the same locus do not correspond to a single sequencing direction, and currently, Stacks does not perform reverse‐complement comparison. Following the procedure of Paris et al. (2017), the minimum number of reads to create a stack, the number of mismatches allowed between stacks, and the number of mismatches allowed between sample loci when generating the catalog were respectively determined as 3, 4, and 5 for *L. boringii* while as 3, 5, and 6 for *Q. boulengeri* . These settings maximized the number of polymorphic loci present in 80% of the individuals.

The Stacks output biallelic SNPs were first filtered with VCFtools version 0.1.16 (Danecek et al. 2011) by keeping genotypes with a read depth ≥ 6 and a genotyping quality ≥ 30, and by removing SNPs present in < 80% of the individuals. TBtools‐II version 2.096 (Chen et al. 2023) was used to extract the consensus sequence for each retained locus from the Stacks output file populations.loci.fa. Then, by using BLAST+ version 2.15.0 (Camacho et al. 2009), with the expectation value cutoff set to 1e‐10, these sequences were compared to the chromosome‐level genome assembly of a closely related congener, that is, *L. leishanense* (GenBank accession GCA_009667805.1; Zheng et al. 2008; Pyron 2014; Li et al. 2019, 2023) or *Q. spinosa* (doi.org/10.5061/dryad.ghx3ffbpw; Yan et al. 2021; Hu et al. 2022). Loci with a unique hit on chromosome‐level scaffolds were kept. They were further blasted using the same settings against a conspecific mitochondrial genome under GenBank accession KJ630505 ( *L. boringii* ; Xu et al. 2016) or KC686711 ( *Q. boulengeri* ; Shan et al. 2014), with those having a hit excluded. Subsequently, loci with the top 1% average read depths were excluded to avoid potential effects of repetitive regions, resulting in the full SNP dataset.

### 2.2 MAF Filtering and Datasets for Uniqueness Estimation

First, for each species, the commonly used MAF filters of different strengths, 1%, 2%, 5%, and 10%, were applied to the full SNP dataset with VCFtools. Then, according to the BLAST hit locations, the first locus from a chromosome and every first locus after 10, 50, or 300 kb were retained to reflect different extents of thinning for reducing the effects of linkage disequilibrium (e.g., Abecasis et al. 2001; Hench et al. 2019; Johri et al. 2021). Lastly, the script randSnps.pl (https://www.biostars.org/p/313701/) was used to randomly select a SNP from each retained locus. This procedure was performed with 10 replicates to account for uncertainty. A total of 300 SNP datasets with a genotype depth of ≥ 6 from the two species were generated to quantify uniqueness.

To represent estimates based on a higher genotype depth, datasets with ≥ 10 depth were also generated from the full SNP dataset with no or 5% MAF filtering and > 50 kb spacing while keeping all other settings constant.

To explore the impact of the percentage of the most genetically distinct samples, the *L. boringii* site L01 with 7 individuals (Table S1) was excluded to simulate a sampling with a lower percentage of such samples. Five representative datasets of this species were generated from loci of ≥ 6 genotype depth, and > 50 kb spacing, with 1%, 2%, 5%, 10%, or no MAF filtering and with one randomly selected SNP per locus.

### 2.3 Uniqueness Quantification Based on Between‐Individual Distances

For each dataset, with pairwise deletion of missing SNPs, genetic distances between individuals were calculated with the widely used Euclidean and Hamming methods by dartR version 2.9.7 (Mijangos et al. 2022) and PLINK version 1.90b6.5 (Chang et al. 2015), respectively. When applied to biallelic SNPs as in this study, the Hamming distance is identical to the frequently used Manhattan distance.

The commonly used 0.5‐degree resolution in biodiversity studies was adopted (e.g., Isbell et al. 2015; Venegas‐Li et al. 2019). For each individual, the distances to individuals from other 0.5‐degree grid cells were averaged first by locality, then by grid cell, and lastly across grid cells to obtain a single individual‐level value. The obtained values were averaged by locality, and the highest resulting mean value within a grid cell was taken as an estimate of uniqueness for that grid cell, that is, how unique it could be. These intraspecific uniqueness estimates were normalized using min‐max scaling (subtracting the minimum and dividing by the range), and averages across the 10 replicates were calculated. For each grid cell, with all other parameters being equal, the replicate estimates from data with or without MAF filtering were compared using unpaired *t*‐, Welch's *t*‐, or Mann–Whitney *U* tests. Normality and equal variances were first checked with the Shapiro–Wilk test and Levene's test, respectively. An unpaired *t*‐test was used if the null hypotheses of normality for both sets of estimates and equal variances were not rejected at 5% level. Welch's *t*‐test was used if only equal variances were rejected. The Mann–Whitney *U* test was performed when the normality of at least one set of estimates was rejected. A paired test across grid cells was not used because different effects of MAF filtering on uniqueness estimates, for example, an increase or decrease, were observed among cells. Besides, to assess whether grid cells with many private alleles were more likely to be less differentiated after filtering, a custom script (privateA2.py) was written to calculate the number of private alleles for each cell from unfiltered data. It was applied to the representative data of ≥ 6 genotype depth and > 50 kb spacing in both species.

The normalized intraspecific estimates were combined by adding when a grid cell was shared by two species to obtain an across‐species estimate. For a given combination of MAF filtering, physical spacing, and distance method, each time one replicate was randomly selected from each species to calculate across‐species estimates for individual shared grid cells. This procedure was repeated 1000 times, and the mean and standard deviation (SD) of these estimates were calculated. For each shared grid cell, assuming repeated comparisons based on random selection and keeping all other parameters constant, such 1000 values from data with or without MAF filtering were compared by paired *t*‐ or Wilcoxon signed‐rank tests. Normality of the differences was first checked using D′Agostino's K‐squared test. When it was rejected at 5% level, a Wilcoxon signed‐rank test was performed; otherwise, a paired *t*‐test was used.

## 3 Results

### 3.1 SNP Data

**FIGURE 1:**
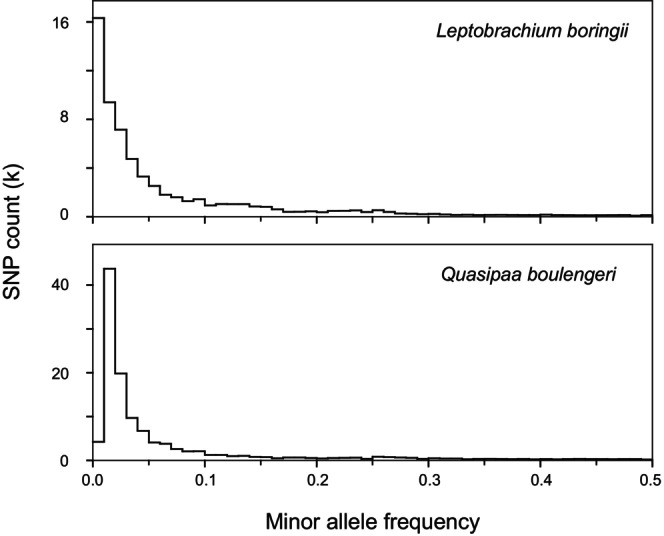
The minor allele frequency distributions of two subsets of loci with genotype depths ≥ 6 and no MAF filtering. Note that one locus was selected every 50 kb based on positions determined from BLAST results.

The full SNP dataset of *Leptobrachium boringii* contained 17,686 loci with a mean genotype depth of 12.0, and it was 86% complete; the *Quasipaa boulengeri* full SNP dataset comprised 37,493 loci with a mean depth of 14.8 and was 84% complete. These loci showed unique BLAST hits from throughout the genomes of congeners. After MAF filtering and physical spacing, 11,228 to 4576 loci from the full dataset of *L. boringii* , with an average of 6.7 to 2.1 SNPs per locus, were used to generate replicate datasets for uniqueness estimation. The *Q. boulengeri* data subsets used had 21,140 to 5360 loci and an average of 7.6 to 2.1 SNPs per locus (Table 1). The subsets with ≥ 10 genotype depth contained less data, 980 or 923 loci for *L. boringii* and 1910 or 1687 loci for *Q. boulengeri* . For both species, most SNPs had a MAF below 5%, and approximately 40% of the SNPs showed MAFs below 2% (Figure 1). The full and replicate datasets are deposited in ScienceDB (https://doi.org/10.57760/sciencedb.16715).

**TABLE 1:** Number of loci (italic) and number of SNPs per locus (mean with SD in parentheses) obtained using MAF and spacing filters.

Spacing	*Leptobrachium boringii*					*Quasipaa boulengeri*				
	C	MAF 0.01	MAF 0.02	MAF 0.05	MAF 0.10	C	MAF 0.01	MAF 0.02	MAF 0.05	MAF 0.10
> 10 kb	*11,228*	*11,037*	*10,816*	*10,011*	*8711*	*21,140*	*21,120*	*20,454*	*17,444*	*13,878*
	6.7 (3.7)	5.1 (3.1)	4.2 (2.7)	2.9 (1.9)	2.2 (1.4)	7.6 (4.5)	7.3 (4.4)	4.7 (3.3)	2.8 (2.2)	2.1 (1.6)
> 50 kb	*9561*	*9417*	*9262*	*8657*	*7655*	*15,543*	*15,530*	*15,181*	*13,484*	*11,272*
	6.7 (3.7)	5.1 (3.1)	4.2 (2.7)	2.9 (1.8)	2.2 (1.4)	7.6 (4.6)	7.4 (4.4)	4.7 (3.3)	2.8 (2.2)	2.1 (1.6)
> 300 kb	*5290*	*5244*	*5203*	*4972*	*4576*	*6264*	*6263*	*6196*	*5871*	*5360*
	6.6 (3.7)	5.0 (3.1)	4.2 (2.6)	2.8 (1.8)	2.1 (1.3)	7.7 (4.7)	7.5 (4.5)	4.8 (3.4)	2.8 (2.2)	2.1 (1.6)

*Note:* Genotype read depth ≥ 6. C: MAF filter not applied; kb: kilobase.

### 3.2 Intraspecific Uniqueness Estimates

**FIGURE 2:**
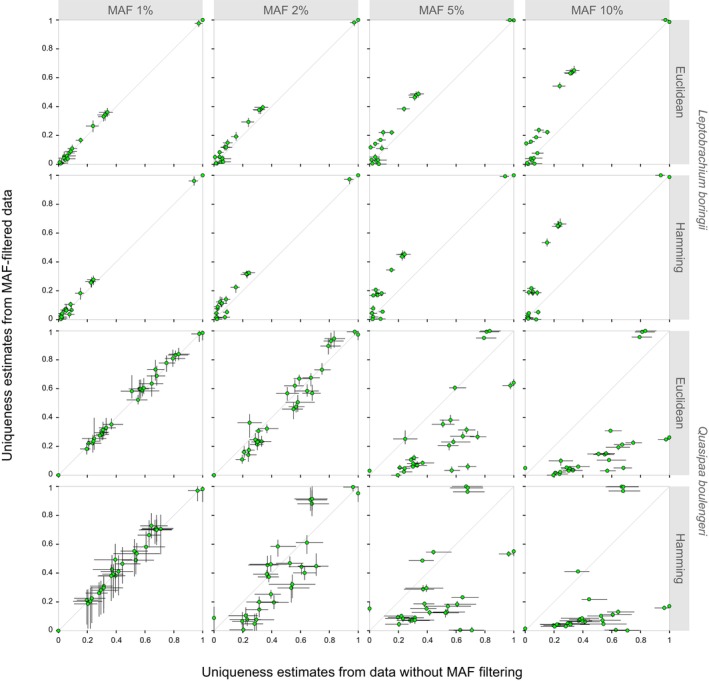
Comparison of normalized intraspecific uniqueness estimates for 0.5‐degree grid cells from data with or without MAF filtering, based on Euclidean and Hamming distances between individuals; note that one SNP was selected every 50 kb based on positions determined from BLAST results. Means of 10 replicate estimates were used to create scatter plots. Bars are ranges. SNPs were genotyped at read depths ≥ 6.

Using different distance methods, spacing settings, and minimum genotype depths, similar effects of MAF filtering on uniqueness estimates were observed. For individuals from the same locality, similar average distances to samples from other 0.5‐degree grid cells were obtained, with for instance a mean coefficient of variation (CV) of 0.72% (range 0.04%–2.32%, *n* = 24) for replicate means from *L. boringii* datasets with ≥ 6 depth, no MAF filtering, and > 50 kb spacing when Euclidean distance was used. In *Q. boulengeri* , the corresponding mean and range of CV were 0.68% and 0.02%–2.26% (*n* = 14). Using these representative datasets, no correlation was detected between the average distance to other grid cells' samples and the genotyping rate or depth of an individual (Spearman's test, all *p* > 0.05). Based on Euclidean or Hamming distances, the unnormalized estimates for 0.5‐degree grid cells ranged up to approximately 1.31–1.41 or 1.55–1.78 times the minimum for *L. boringii* and about 1.22–1.40 or 1.24–1.73 times in *Q. boulengeri* , respectively. The plots between normalized estimates from SNPs with ≥ 6 depth and > 50 kb spacing are presented in Figure 2 as an example, and those based on a ≥ 10 genotype depth or > 10 and > 300 kb spacings are shown in Figures S1–S3. In both species, the deviation of the results from those obtained from data without MAF filtering increased with the MAF threshold, despite higher variation for replicate datasets with ≥ 10 depth. Generally, grid cells that were less differentiated after filtering had more private alleles (Figure S4).

In *L. boringii* , when using all samples, the high‐uniqueness grid cells comprised the two southernmost ones in all cases. The deviation was slight at thresholds of 1% and 2% and showed the most pronounced increases for middle‐uniqueness grid cells at 5% and 10% (Table S2). For instance, with the Euclidean method, ≥ 6 depth, and > 50 kb spacing, one hotspot‐edge grid cell (at the eastern edge of the Mountains of Southwest China hotspot) received average normalized estimates of 0.313, 0.331, 0.376, 0.463, and 0.630 at no threshold or 1%, 2%, 5%, and 10% thresholds, respectively. When one of the most unique local populations (L01) was excluded, three hotspot‐edge grid cells were observed to replace the southernmost cell as the most unique ones at thresholds 5% and 10% (Figure S5).

In *Q. boulengeri* , the grid cells with the highest estimates remained the same at 1% and 2%, and they, that is, the easternmost two, were replaced by three hotspot‐edge grid cells at 5% and 10%. The deviation was slight at 1% but showed notable decreases for some grid cells at 2% and for most grid cells at higher thresholds (Table S3). For example, when Hamming distance, ≥ 6 depth, and > 50 kb spacing were applied, average normalized estimates of 0.709, 0.705, 0.447, 0.003, and 0.001 were, respectively, obtained at no threshold or 1%, 2%, 5%, and 10% thresholds for one of the southernmost grid cells.

### 3.3 Across‐Species Uniqueness Estimates

**FIGURE 3:**
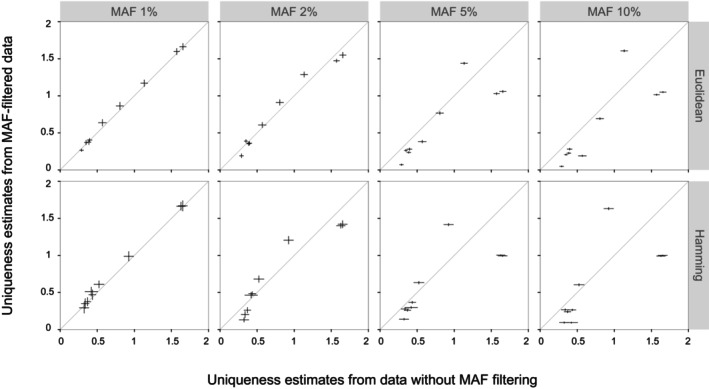
Comparison of across‐species uniqueness estimates of *Leptobrachium boringii* and *Quasipaa boulengeri* for 0.5‐degree grid cells from data with or without MAF filtering, based on the Euclidean and Hamming distances between individuals; note that one SNP was selected every 50 kb based on positions determined from BLAST results. Normalized intraspecific uniqueness estimates were combined by addition. Means of 1000 replicate estimates were used to create scatter plots. Error bars are SDs. SNPs were genotyped at read depths ≥ 6.

**FIGURE 4:**
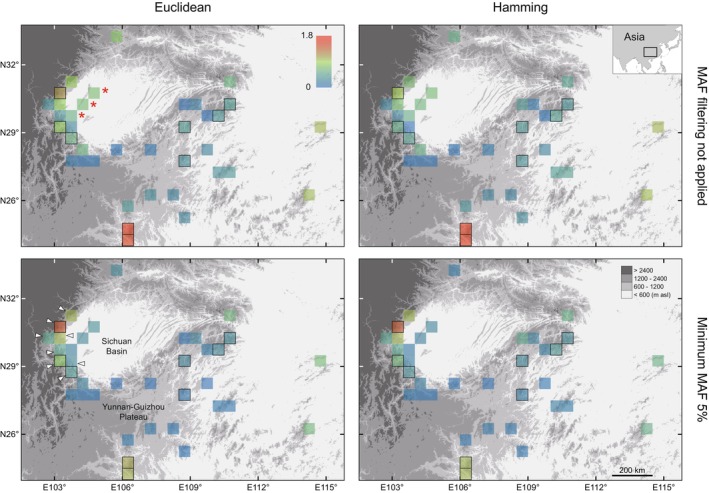
Uniqueness estimates from data with 5% MAF filtering or without MAF filtering, based on Euclidean and Hamming distances between individuals; note that one SNP was selected every 50 kb based on positions determined from BLAST results. Mean values of replicate estimates were used. SNPs were genotyped at read depths ≥ 6. Grid cells with black outlines are shared by the two species, *Leptobrachium boringii* and *Quasipaa boulengeri* . Normalized intraspecific uniqueness estimates were combined by addition for each shared grid cell. Grid cells representing *Q. boulengeri* localities along a mountain range within the basin are marked by an asterisk. Grid cells at the eastern edge of the Mountains of Southwest China biodiversity hotspot are indicated by arrows.

Similar effects of MAF filtering on estimates across the two species were observed for different distance methods, physical spacings, and minimum read depths. Based on Euclidean distance, the mean estimates for each of the nine shared grid cells ranged from 0.048–0.358 to 1.367–1.796, and by Hamming distance, the means ranged from 0.086–0.323 to 1.333–1.825. The plots comparing estimates from SNPs with ≥ 6 depth and > 50 kb spacing are given in Figure 3, with those based on a ≥ 10 depth or > 10 and > 300 kb spacings in Figures S3 and S6. Again, the estimates' deviation from those obtained with no MAF filtering increased with the MAF threshold. It was statistically significant for individual grid cells in most cases (Table S4), being slight at 1% and 2%, but considerable for some grid cells at higher thresholds. As a result, one hotspot‐edge grid cell eventually replaced the southernmost two as the grid cell with the highest estimate (Figures 4 and S7). For instance, based on the Hamming distance and with ≥ 6 depth and > 50 kb spacing applied, mean values of 0.924, 0.988, 1.206, 1.416, and 1.632 were, respectively, obtained for the former at no threshold or 1%, 2%, 5%, and 10% thresholds, while corresponding values of 1.629, 1.665, 1.402, 1.004, and 0.993 were obtained for one of the latter.

## 4 Discussion

### 4.1 Effect of MAF Filtering on Uniqueness Estimates

Minor allele filtering can considerably influence estimates of uniqueness. At both within‐ and across‐species levels, the difference between estimates derived from data with or without MAF filtering increased with the threshold of MAF, being significant mostly at thresholds of 5% and 10% (Figures 2 and 3). This pattern is in accordance with a gradual increase of bias, and several factors support it. First, as individuals from the same locality showed similar average distances to samples in other grid cells, and as no correlation between the distance and genotype depth had been detected, a high uniqueness was not likely due to sequencing errors that affected genotyping more at lower read depths, indicating the reliability of estimates from unfiltered data as references. Second, the effect of sample size, that is, fewer loci at higher MAF thresholds, should be negligible. In both species, the assumed > 300 kb spacing resulted in the smallest numbers of loci (Table 1) but yielded similar mean estimates when compared to the > 10 and > 50 kb ones. Last, the estimates based on unfiltered data seem intuitive. High uniqueness had been estimated for the southern, eastern, and northern insular grid cells and for those representing an isolated mountain range within the Sichuan basin (Figure 4), both of which had been shown to harbor significant endemic clusters (Zheng et al. 2020, 2025). However, MAF thresholds of 5% and 10% could cause a substantial decrease in estimates for these grid cells while clearly turning a hotspot‐edge grid cell into the one with the highest estimate (Figures 2, 3, 4). This grid cell is unique among the three hotspot‐edge grid cells shared by the two species due to having samples of a lineage endemic to the edge from both species (Zheng et al. 2020, 2025). It received increased estimates in both species, probably because those loci showing the rarest alleles elsewhere had been filtered. Taken together, it is reasonable to consider that the present results demonstrate that MAF filtering can cause both dramatic underestimation and overestimation of uniqueness.

The effect of an overall MAF filter may depend partly on the percentage of samples that are most genetically distinct. If the percentage is below the filtering threshold, the filtering may make such samples appear less unique by removing loci with rare alleles specific to them. In *Leptobrachium boringii* , such samples comprised ten individuals from the two close southernmost localities (Table S1). The percentage is 13.3%, so a significant number of loci supporting their uniqueness should have passed the strictest MAF filter here, 10%. In fact, the high‐uniqueness grid cells remained unchanged when using all samples. After excluding one of the two localities, this percentage dropped to 4.4%, and at the MAF threshold of 5%, three hotspot‐edge grid cells began to replace the remaining southernmost cell as the high‐uniqueness ones (Figure S5). However, after filtering, a high percentage of samples ranked as the most distinct does not guarantee the reliability of the highest estimates. In *Quasipaa boulengeri* , the most unique samples comprised four individuals from the two easternmost localities ~330 km apart, two individuals (3.6%) each. They were replaced by 13 individuals (23.6%) from six localities within three adjacent hotspot‐edge grid cells at MAF thresholds of 5% and 10%, although not at 2% or 1%. If a dataset has an extremely low proportion of the most unique samples, for instance, below 1%, applying a minimum MAF of 2% or 1% will possibly also hinder uniqueness estimation.

The estimates for *Q. boulengeri* (Figure 2) imply that a relatively unique local population can appear to be genetically typical at higher MAF thresholds. Actually, with the Hamming distance applied, the use of a MAF filter of 5% or 10% caused the mean of normalized replicates for one of the two southernmost localities to drop from approximately 0.65–0.85 to a minimum close to 0 in all cases (Table S3). Thus, it is possible that minor allele filtering may hinder the detection of the most typical/representative populations when using genetic distances.

The use of *L. boringii* and *Q. boulengeri* allows the identification of a long‐distance (~750 km) shift of the region with the highest across‐species uniqueness estimate caused by MAF filtering. One may suspect that such significant impacts are likely to be observed only in simple systems with two or a few species due to severely biased intraspecific estimates in the same regions by chance. This needs to be confirmed by further studies since the genetic structures of different species have often been strongly shaped by the same factors (e.g., Martins et al. 2022; Dapporto et al. 2024; Marske and Boyer 2024).

On the other hand, uniqueness estimates generally increased in *L. boringii* and decreased in *Q. boulengeri* at higher MAF thresholds (Figures 2 and S5). This also supports that the impacts of MAF filtering on uniqueness estimates are not necessarily consistent across taxa. In both species, grid cells that became less unique after filtering in general had more private alleles (Figure S4). Thus, the difference may be partly explained by most grid cells having many private and rare alleles in *Q. boulengeri* but not in *L. boringii* due to different demographic histories. The wider range of estimates from replicate datasets in *Q. boulengeri* (Figure 2) is possibly also partly related to demographic histories. These replicate datasets were generated by randomly selecting one SNP from each locus, exhibiting non‐identical distributions of rare alleles among local populations. A locus can have different SNP‐specific rare alleles occurring in different populations. If this is much more prevalent in *Q. boulengeri* than in *L. boringii* , more variation between replicate datasets may contribute to a wider range of estimates in the former when using no or low MAF thresholds. In both species, the range became narrower at higher MAF thresholds. Long‐term demographic inferences for five *L boringii* populations suggest periodic fluctuations (Fu and Wen 2023; Li et al. 2023), while comparable data are lacking for *Q. boulengeri* . Besides, at the same MAF threshold, estimates based on datasets containing more SNPs generally spanned a narrower range in both species (Table 1; Figures 2 and S1–S3), implying an effect of sample size.

### 4.2 Implications of the Findings

The current results are in line with previous studies showing substantial effects of minor allele filtering on downstream analyses such as demographic, population structure, and phylogenomic inferences (Linck and Battey 2019; Hemstrom et al. 2024; Rick et al. 2024). Given the fact that low‐frequency variants are often prevalent in empirical data (e.g., Hall et al. 2020; Marandel et al. 2020; Fu et al. 2021; Clark et al. 2022) and that young (Linck and Battey 2019), local (la Cruz and Raska 2014), introgressed, or rare alleles all can be infrequent in a dataset, minor allele filtering may remove not only a large portion of true data but also information critical to inferring some particular aspects of species history. This is not desired, at least for uniqueness quantification. However, the use of minor allele filtering instead of genotype quality score and depth filtering to minimize potential genotyping errors may be considered a high priority, such as when working on ancient genomes with postmortem damage and low sequencing coverage (Kousathanas et al. 2017). If this is the case, one may apply both no minor allele filter and an often‐used overall minor allele count filter of ≥ 3, which is less stringent but requires an allele occurring in more than one diploid individual (O'Leary et al. 2018; Schmidt et al. 2021; Hemstrom et al. 2024). When the results are compared, it is still possible to find different most unique local populations if each locality usually has one or two individuals sampled. In the analysis of sex‐linked SNPs, where minor allele filtering is widely employed (e.g., Hill et al. 2018; Evans et al. 2024; Yi et al. 2024), applying a minimum minor allele count of 3 has been reported to have no or little effect on the results (Robledo‐Ruiz et al. 2025). On the other hand, a MAF filter might exclude rare, often new sex‐linked alleles with sample occurrences sufficient for a statistical inference (Trenkel et al. 2020; Evans et al. 2024).

For uniqueness estimation, one may consider first applying mapping quality, genotype depth, and genotype quality filters to minimize genotyping errors and then quantifying and reporting the effect of a study‐wide minor allele count filter of ≥ 3 (Hemstrom et al. 2024). If the impact is not negligible for the question of interest, it can prompt efforts to avoid relying on an inappropriate dataset.

The quantification of biodiversity and conservation planning involve the integration of various variables. Although the contribution of individual variables may vary substantially according to the context, it is important to first obtain unbiased estimates for each of them. Some factors may significantly impact the quantification/planning by affecting the estimates of more than one variable. Minor allele filtering is likely one such factor. It has been shown to be able to considerably bias the estimates of heterozygosity (Schmidt et al. 2021; Arantes et al. 2023) and effective population size (Marandel et al. 2020). The findings in this study make uniqueness another such variable. They are, however, based on a simple uniqueness estimation approach using average genetic distances without controlling for sample size. The approach may be one important choice for many datasets with typically a few individuals per locality, but it may be less useful when the sampling is adequate for more advanced methods. Hence, these findings suggest not only caution in data processing and result interpretation but also further efforts on the effect of minor allele filtering on uniqueness estimates, preferably with relatively universal markers such as a conservative subset of the exome (Waterhouse et al. 2018) and with simulation‐based validation. For example, simulations will likely help us better understand how universal MAF filtering affects spatial genetic distance estimates.

## 5 Conclusion

This study provides early evidence for dramatic effects of MAF filtering on within‐ and across‐species estimates of genetic uniqueness, which call for caution and further investigation. Minor allele filtering may bias the quantification of biodiversity and conservation planning by affecting the estimates of multiple contributing variables. While the construction of high‐throughput sequencing datasets is fast developing, the findings here are among those supporting that the effects of many involved parameters and filters on downstream analyses remain to be better understood.

## Author Contributions

Yuchi Zheng: conceptualization, funding acquisition, data curation, formal analysis, visualization, writing – original draft, writing – review and editing.

## Funding

This work was supported by the National Natural Science Foundation of China, 32170465.

## Conflicts of Interest

The author declares no conflicts of interest.

## Supporting information


**Data S1:** supporting Information

**Table S2:** Comparison of replicate normalized uniqueness estimates with or without minor allele frequency (MAF) filtering for each 0.5‐degree grid in *Leptobrachium boringii* . *n* = 10 for each variable. *P* values > 0.05 were marked by gray background. U: Mann–Whitney U test.

**Table S3:** Comparison of replicate normalized uniqueness estimates with or without minor allele frequency (MAF) filtering for each 0.5‐degree grid in *Quasipaa boulengeri* . *n* = 10 for each variable. *P* values > 0.05 were marked by gray background. U: Mann–Whitney U test.

**Table S4:** Comparison of uniqueness estimates with or without minor allele frequency (MAF) filtering for each 0.5‐degree grid shared by *Leptobrachium boringii* and *Quasipaa boulengeri*, assuming repeated comparisons based on randomly selected replicates. *n* = 1000 for each test. *P* values > 0.05 are shown in bold. Wilcoxon: Wilcoxon signed‐rank test.

## Data Availability

The data underlying this article including the 340 replicate datasets used to quantify uniqueness, the two full SNP datasets, the five datasets simulating a sampling with a lower percentage of the most genetically distinct samples, all genetic distance and uniqueness estimates, and custom Python scripts for statistical tests or calculating private alleles are available on ScienceDB (https://doi.org/10.57760/sciencedb.16715).
