# Supplementary material for: Minor Allele Frequency Filtering Can Strongly Bias Uniqueness Estimates Within and Across Species
Source: Ecol Evol. 2026 Mar 24;16(3):e73314. doi: 10.1002/ece3.73314 (PMC13093744; doi:10.1002/ece3.73314)
Supplement: Supplementary file 1 — Data S1: supporting Information [file ECE3-16-e73314-s004.pdf]

Table S1. Details of sampling localities. *n* : number of individuals.

Species	Locality	Coordinates	*n*
Leptobrachium boringii	L01: Cenwanglaoshan, Guangxi	N24.41°, E106.38°; N24.42°, E106.38°	7
	L02: Cenwanglaoshan, Guangxi	N24.51°, E106.44°	3
	L03: Changyang, Yichang, Hubei	N30.28°, E110.78°	3
	L04: Hupingshan, Hunan	N30.10°, E110.82°	3
	L05: Hupingshan, Hunan	N30.03°, E110.57°	3
	L06: Badagongshan, Hunan	N29.79°, E110.09°	2
	L07: Badagongshan, Hunan	N29.69°, E110.07°	2
	L08: Badagongshan, Hunan	N29.76°, E109.86°	3
	L09: Enshi, Hubei	N30.48°, E109.45°	3
	L10: Youyang, Chongqing	N29.31°, E108.98°	2
	L11: Xianfeng, Hubei	N29.38°, E108.94°	4
	L13: Fanjingshan, Guizhou	N27.90°, E108.72°	3
	L14: Fanjingshan, Guizhou	N27.92°, E108.65°	3
	L15: Suiyang, Guizhou	N28.33°, E107.17°	3
	L16: Gulin, Sichuan	N28.12°, E105.78°	3
	L17: Zhenxiong, Zhaotong, Yunnan	N27.60°, E104.75°	3
	L18: Junlian, Sichuan	N27.98°, E104.43°	1
	L19: Daguan, Yunnan	N27.73°, E103.92°	3
	L20: Muchuan, Sichuan	N28.94°, E103.83°	3
	L21: Shaxi, Sichuan	N29.44°, E103.41°; N29.45°, E103.41°	2
	L23: Omei Mt., Sichuan	N29.57°, E103.39°; N29.60°, E103.39°; N29.60°, E103.38°	4
	L25: Jiajiang, Sichuan	N29.77°, E103.40°	3
	L26: Bifengxia, Yaan, Sichuan	N30.08°, E102.98°	3
	L27: Dayi, Sichuan	N30.72°, E103.41°	3
	L28: Qingchengshan, Sichuan	N30.93°, E103.47°	3
Quasipaa boulengeri	Q03: Kangxian, Gansu	N33.14°, E105.69°	1
	Q05: Pengzhou, Sichuan	N31.24°, E103.81°	1
	Q06: Dujiangyan, Sichuan	N31.09°, E103.61°; N31.10°, E103.63°	2
	Q07: Dayi, Sichuan	N30.68°, E103.45°	2
	Q08: Dayi, Sichuan	N30.63°, E103.38°	1
	Q09: Qionglai, Sichuan	N30.31°, E103.25°; N30.32°, E103.27°; N30.32°, E103.23°	5
	Q10: Yaan, Sichuan	N30.12°, E103.05°	2
	Q11: Jintang, Sichuan	N30.75°, E104.51°	2
	Q12: Chengdu, Sichuan	N30.37°, E104.15°	1
	Q13: Qingshen, Sichuan	N29.76°, E103.85°	1
	Q14: Leshan, Sichuan	N29.34°, E103.53°	1
	Q15: Ebian, Sichuan	N29.11°, E103.22°	2
	Q16: Muchuan, Sichuan	N28.93°, E103.83°	1
	Q17: Muchuan, Sichuan	N28.83°, E103.89°	1
	Q18: Shuifu, Yunnan	N28.43°, E104.11°	1
	Q28: Lichuan, Hubei	N30.41°, E108.56°	1
	Q31: Xingshan, Hubei	N31.25°, E110.61°	4
	Q32: Changyang, Hubei	N30.28°, E110.78°	1
	Q35: Xianfeng, Hubei	N29.41°, E108.98°	1
	Q36: Youyang, Chongqing	N29.31°, E108.98°	1
	Q37: Hongjiang, Hunan	N29.505°, E110.17°	2
	Q38: Baojing, Hunan	N28.49°, E109.77°	2
	Q39: Yinjiang, Guizhou	N27.92°, E108.65°	1
	Q40: Jiangkou, Guizhou	N27.89°, E108.73°	1
	Q42: Ziyun, Guizhou	N25.72°, E106.15°	2
	Q43: Guiding, Guizhou	N26.36°, E107.27°	1
	Q44: Guiding, Guizhou	N26.37°, E107.31°	1
	Q45: Leishan, Guizhou	N26.40°, E108.29°	2
	Q46: Tianlin, Guangxi	N24.47°, E106.36°	1
	Q47: Tianlin, Guangxi	N24.51°, E106.44°	1
	Q48: Rongshui, Guangxi	N25.45°, E108.78°	1
	Q49: Hongjiang, Hunan	N27.18°, E110.28°	2
	Q50: Longhui, Hunan	N27.40°, E110.74°	1
	Q51: Longhui, Hunan	N27.45°, E110.82°	1
	Q52: Jinggangshan, Jiangxi	N26.497°, E114.08°; N26.49°, E114.08°	2
	Q53: Tongshan, Hubei	N29.44°, E114.68°	2

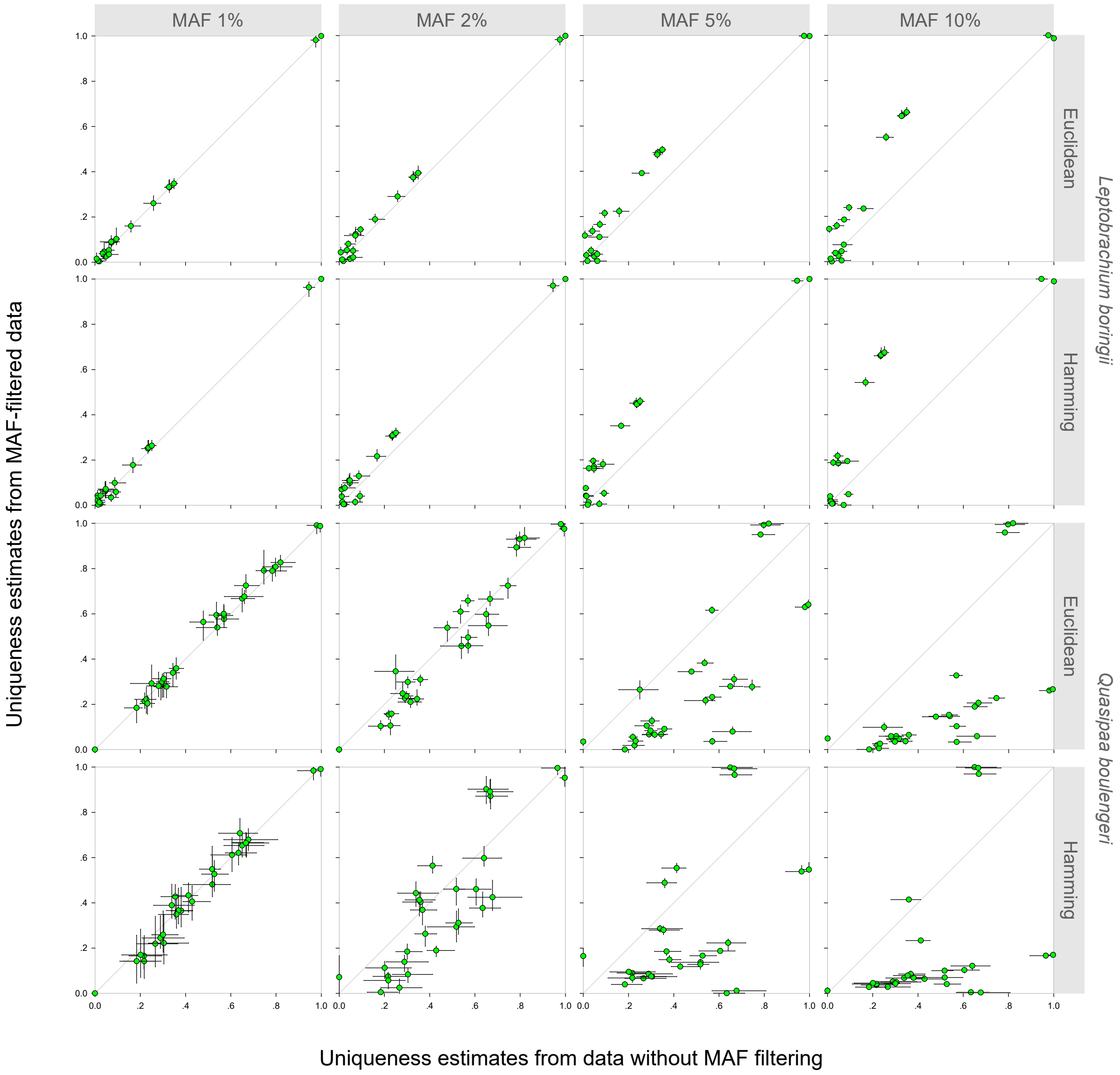

Figure S1. Comparison of normalized intraspecific uniqueness estimates for 0.5-degree grid cells from data with or without minor allele frequency (MAF) filtering, based on Euclidean and Hamming distances between individuals; note that one SNP was selected every 10 kb based on positions determined from BLAST results. Means of 10 replicate estimates were used to create scatter plots. Bars are ranges. SNPs were genotyped at read depths ≥6.

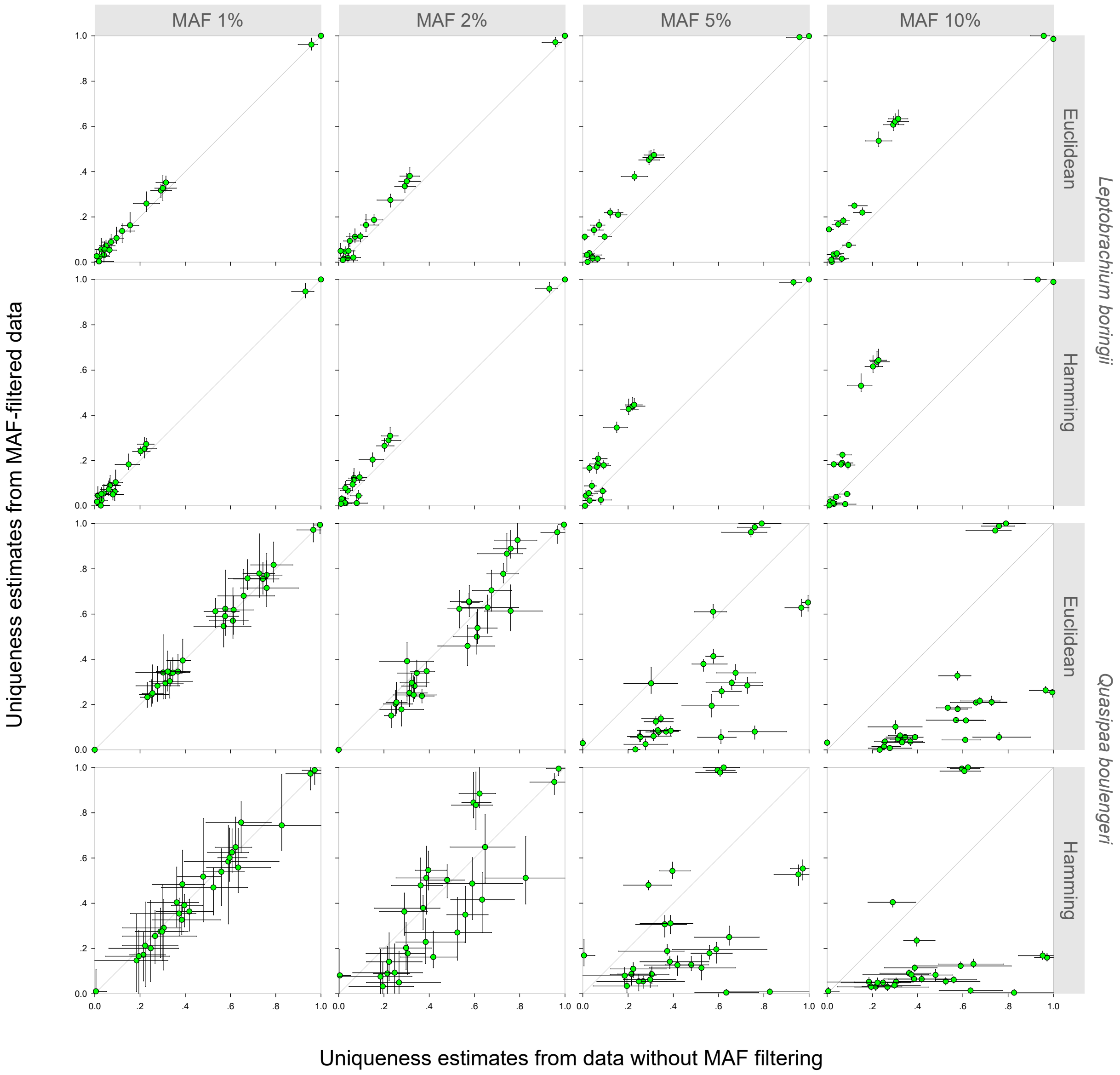

Figure S2. Comparison of normalized intraspecific uniqueness estimates for 0.5-degree grid cells from data with or without minor allele frequency (MAF) filtering, based on Euclidean and Hamming distances between individuals; note that one SNP was selected every 300 kb based on positions determined from BLAST results. Means of 10 replicate estimates were used to create scatter plots. Bars are ranges. SNPs were genotyped at read depths ≥6.

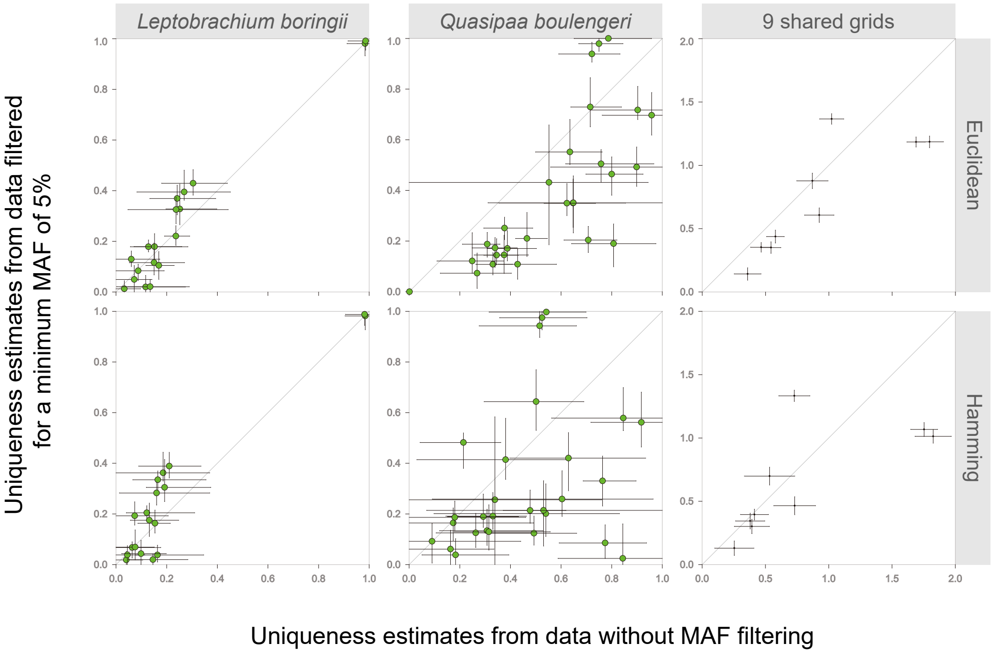

Figure S3. Comparison of intraspecific and across-species uniqueness estimates for 0.5-degree grid cells from data with or without minor allele frequency (MAF) filtering, based on Euclidean and Hamming distances between individuals; note that SNPs were genotyped at read depths ≥10 and one SNP was selected every 50 kb based on positions determined from BLAST results. The two species shared 9 grid cells. Normalized intraspecific uniqueness estimates were combined by adding. Means of replicate estimates were used to create scatter plots. Bars are ranges for intraspecific plots and SD for across-species ones.

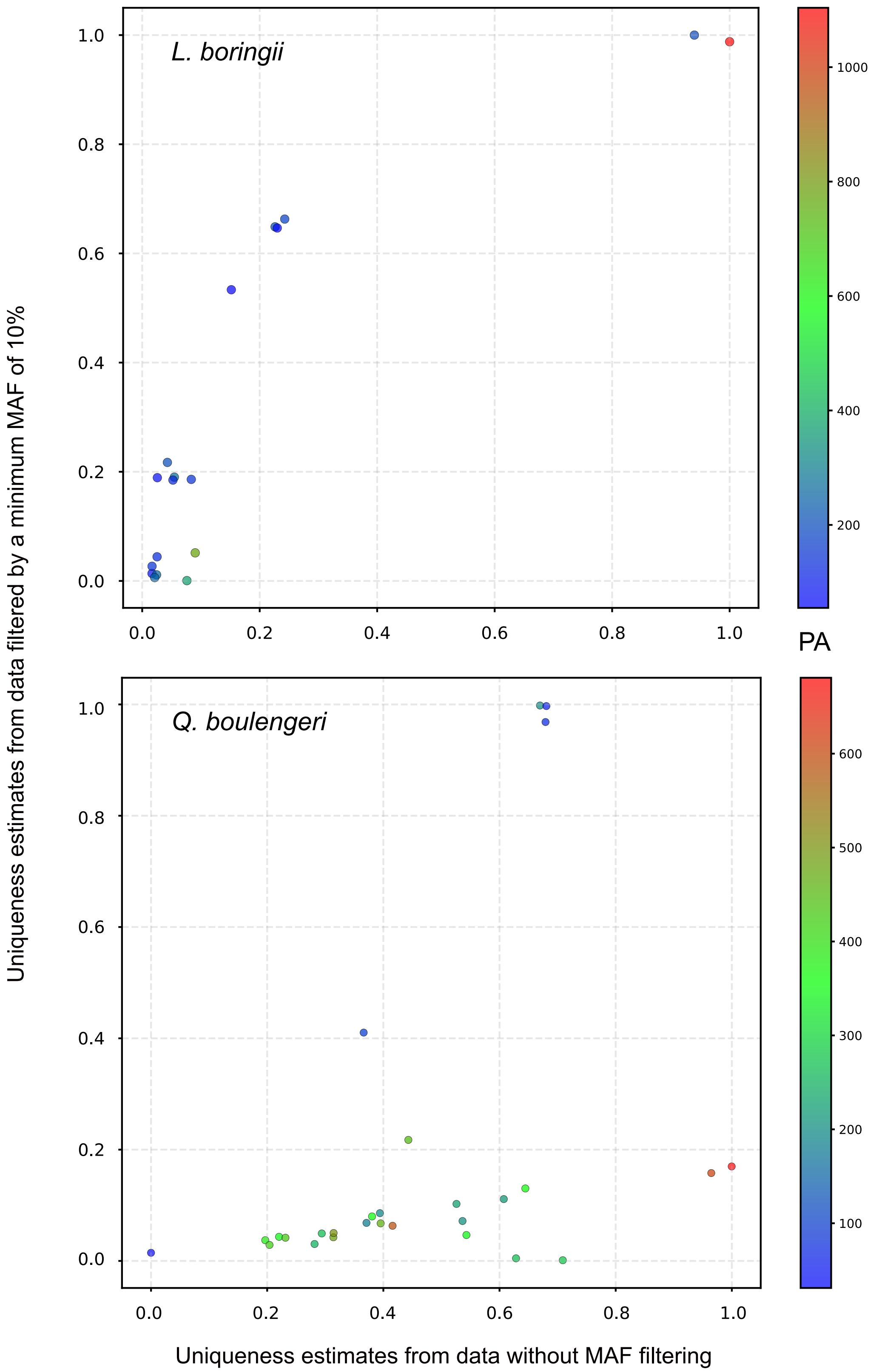

Figure S4. Comparison of normalized intraspecific uniqueness estimates for 0.5-degree grid cells from data with 10% MAF filtering or without MAF filtering, based on Hamming distances between *Leptobrachium boringii* or *Quasipaa boulengeri* individuals; note that one locus was selected every 50 kb based on positions determined from BLAST results. SNPs were genotyped at read depths ≥6. A single SNP was randomly selected from each locus. PA: number of private alleles for each grid cell.

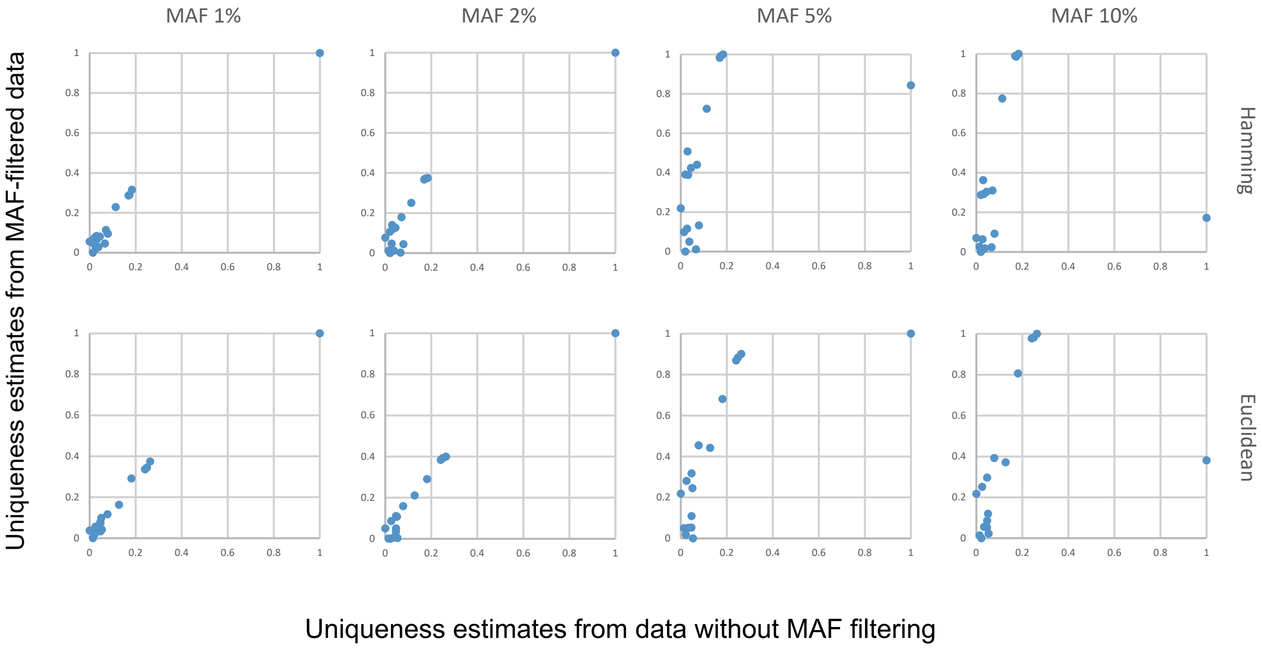

Figure S5. Comparison of normalized intraspecific uniqueness estimates for 0.5-degree grid cells from data with or without MAF filtering, based on Hamming or Euclidean distances between *Leptobrachium boringii* individuals; note that the 7 individuals from the locality L01 were excluded from analysis. SNPs were genotyped at read depths ≥6. One locus was selected every 50 kb based on positions determined from BLAST results. Then MAF filtering was applied. A single SNP was randomly selected from each locus.

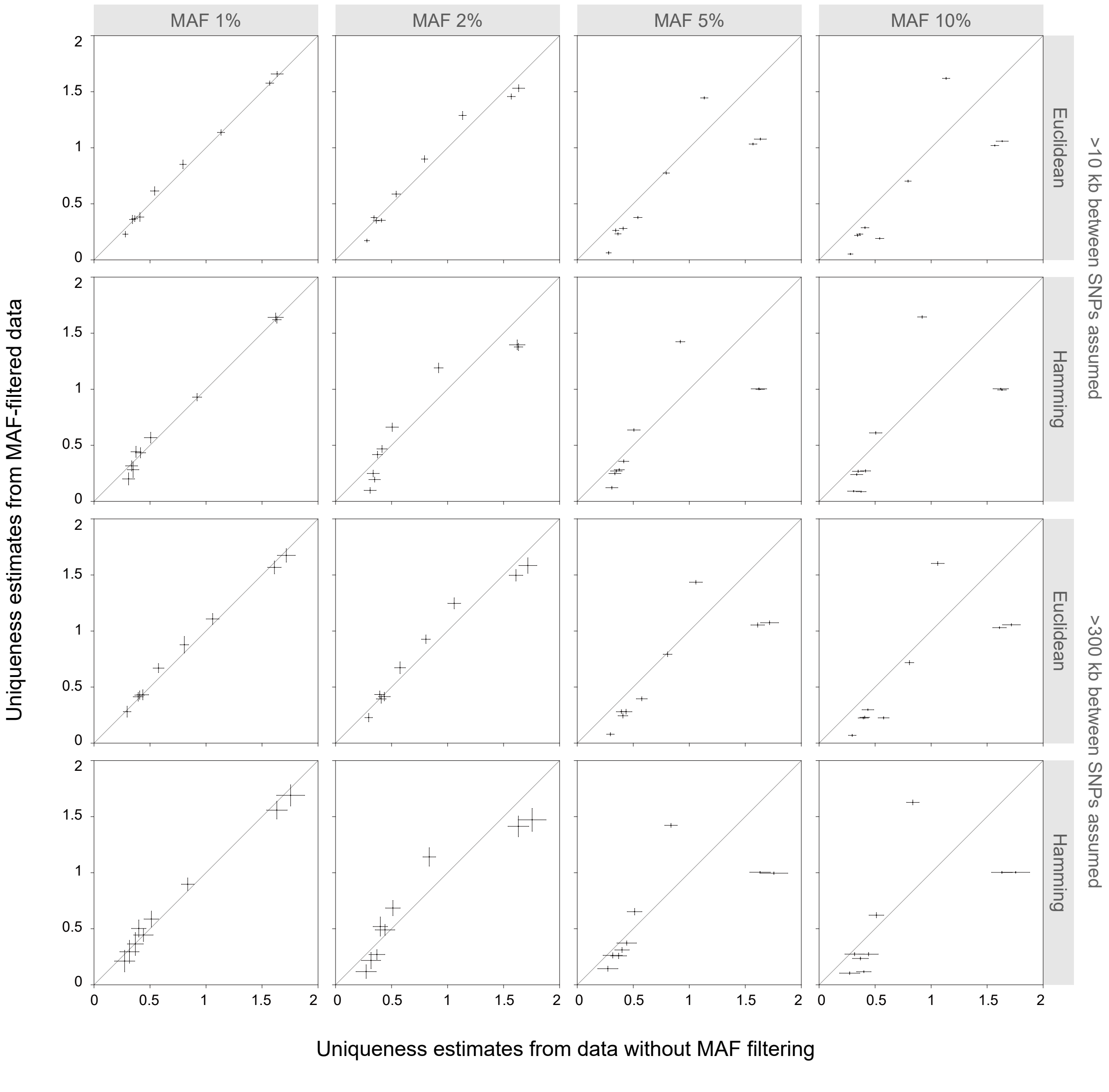

Figure S6. Comparison of uniqueness estimates across *Leptobrachium boringii* and *Quasipaa boulengeri* for 0.5-degree grid cells from data with or without MAF filtering, based on Euclidean and Hamming distances between individuals; note that one SNP was selected every 10 or 300 kb based on positions determined from BLAST results. Normalized intraspecific uniqueness estimates were combined by adding. Means of 1000 replicate estimates were used to create scatter plots. Error bars are SDs. SNPs were genotyped at read depths ≥6.

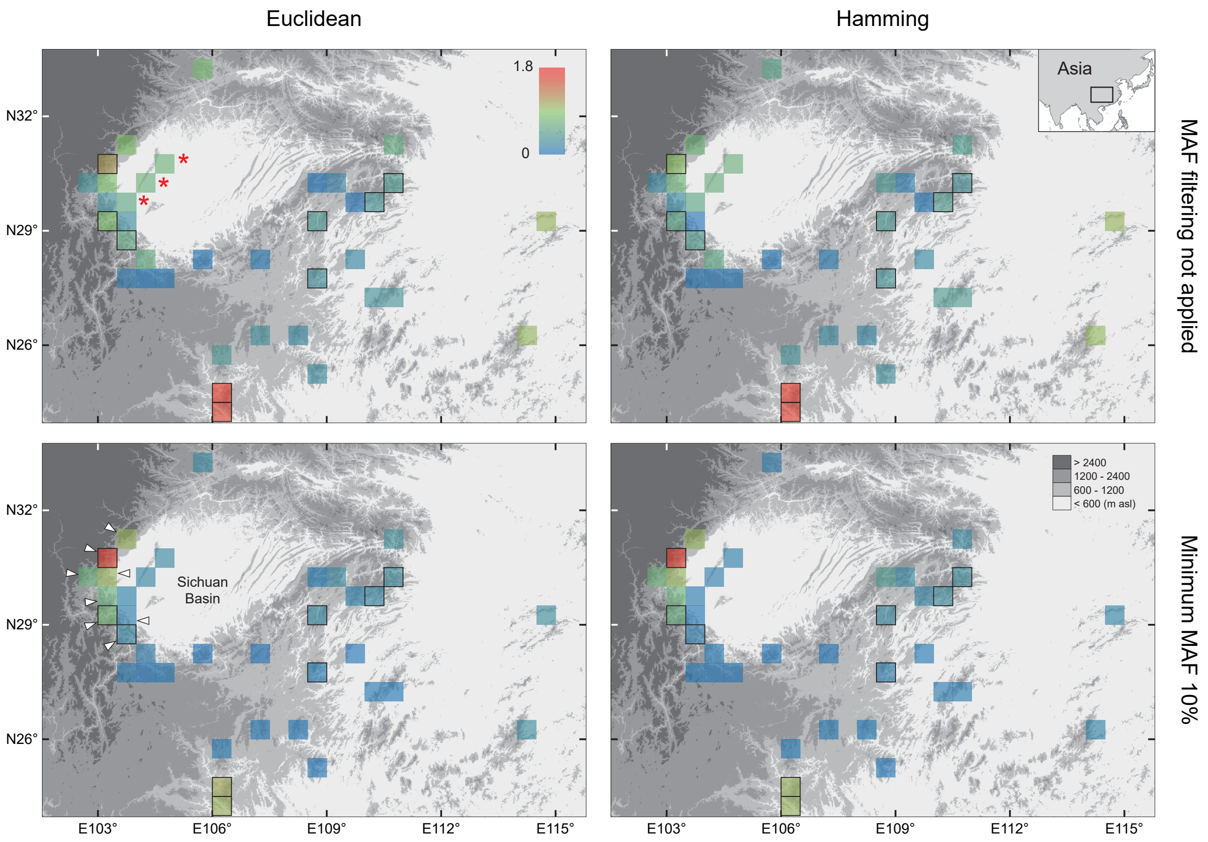

Figure S7. Uniqueness estimates from data with 10% MAF filtering or without MAF filtering, based on Euclidean and Hamming distances between individuals; note that one SNP was selected every 50 kb based on positions determined from BLAST results. Mean values of replicate estimates were used. SNPs were genotyped at read depths ≥6. Grid cells with black outlines are shared by the two species. Normalized intraspecific uniqueness estimates were combined by adding for each shared grid cell. Grid cells representing *Quasipaa boulengeri* localities along a mountain range within the basin are marked by an asterisk. Grid cells at the eastern edge of the Mountains of Southwest China biodiversity hotspot are indicated by arrows.
